# Predictive value of visit-to-visit blood pressure variability for cardiovascular events in patients with coronary artery disease with and without diabetes mellitus

**DOI:** 10.1186/s12933-021-01280-z

**Published:** 2021-04-24

**Authors:** Yuen-Kwun Wong, Yap-Hang Chan, JoJo S. H. Hai, Kui-Kai Lau, Hung-Fat Tse

**Affiliations:** 1 Department of Medicine, The University of Hong Kong, Queen Mary Hospital, Hong Kong, China; 2 Department of Medicine, Shenzhen Hong Kong University Hospital, Shenzhen, China; 3 Hong Kong-Guangdong Joint Laboratory On Stem Cell and Regenerative Medicine, The University of Hong Kong, Hong Kong, China; 4 Shenzhen Institutes of Research and Innovation, The University of Hong Kong, Hong Kong SAR, China

**Keywords:** Blood pressure variability, Coronary artery disease, Risk prediction, Type 2 diabetes mellitus

## Abstract

**Background:**

High blood pressure is a major risk factor for cardiovascular disease. Visit-to-visit blood pressure variability (BPV) has recently been shown to predict cardiovascular outcomes. We investigated the predictive value of BPV for major adverse cardiovascular events (MACE) among patients with coronary artery disease (CAD), with and without type 2 diabetes mellitus (T2DM).

**Methods:**

Patients with stable CAD were enrolled and monitored for new MACE. Visit-to-visit BPV was defined as the coefficient of variation (CV) of systolic and diastolic BP across clinic visits. Multivariable logistic regression analysis was performed to evaluate the association of BPV with MACE. Area under the receiver operating characteristic curve (AUC) was used to assess its predictive ability.

**Results:**

Among 1140 Chinese patients with stable CAD, 192 (17%) experienced a new MACE. In multivariable analyses, the risk of MACE was significantly associated with CV of systolic BP (odds ratio [OR] for highest versus lowest quartile, 3.30; 95% CI 1.97–5.54), and diastolic BP (OR for highest versus lowest quartile, 2.39; 95% CI 1.39–4.11), after adjustment for variables of the risk factor model (age, gender, T2DM, hypertension, antihypertensive agents, number of BP measurements) and mean BP. The risk factor model had an AUC of 0.70 for prediction of MACE. Adding systolic/diastolic CV into the risk factor model with mean BP significantly increased the AUC to 0.73/0.72 (*P* = 0.002/0.007). In subgroup analyses, higher CV of systolic BP remained significantly associated with an increased risk for MACE in patients with and without T2DM, whereas the association of CV of diastolic BP with MACE was observed only in those without T2DM.

**Conclusions:**

Visit-to-visit variability of systolic BP and of diastolic BP was an independent predictor of new MACE and provided incremental prognostic value beyond mean BP and conventional risk factors in patients with stable CAD. The association of BPV in CAD patients without T2DM with subsequent risk for MACE was stronger than in those with T2DM.

**Supplementary Information:**

The online version contains supplementary material available at 10.1186/s12933-021-01280-z.

## Background

Individuals with coronary artery disease (CAD) are at increased risk of recurrent cardiovascular events and death. Intensive risk factor management and lifestyle modification are essential for secondary prevention of cardiovascular disease (CVD) [1, 2]. High blood pressure (BP) is one of the major risk factors for development of CVD. The risk of long-term cardiovascular mortality increases progressively as BP rises above 115/75 mmHg [3]. In addition, clinical trials have reported that intensive BP control in high-risk patients, such as those with previous cardiovascular events, type 2 diabetes mellitus (T2DM) and the elderly, is associated with lower rates of major cardiovascular events and mortality [4–6]. Appropriate management of hypertension to achieve optimal BP control is therefore critically important.

Many of the CVD risk estimation scores include BP as one of the core variables. Nonetheless BP is prone to variation so a risk score that relies on a single measurement may underestimate the true effect of the association [7]. Recent studies have demonstrated that BP variability (BPV) is associated with increased risk of cardiovascular events and mortality in high-risk patients [8–11]. Long-term visit-to-visit BPV has also been shown to be reproducible over time and an important risk factor for CVD, CAD, stroke and mortality [12–14]. It is unknown whether visit-to-visit BPV provides any incremental value for risk prediction in patients with established CAD. Furthermore, previous studies demonstrated that visit-to-visit variability in systolic BP was an independent predictor of macro- and microvascular outcomes and mortality in individuals with T2DM [9, 15], but few reported the risk associated with BPV in individuals without T2DM [16]. Whether visit-to-visit BPV among CAD patients with or without T2DM is associated with future risk for CVD events remains unknown.

In this study, we investigated the association of visit-to-visit BPV with risk for major adverse cardiovascular events (MACE) in CAD patients with and without T2DM. We also evaluated the contribution of BPV to cardiovascular risk prediction beyond mean BP and conventional risk factors.

## Methods

### Study population

Consecutive patients with stable CAD who attended follow-up at the Cardiac Clinic, Queen Mary Hospital, Hong Kong between December 2003 and December 2014 were recruited. CAD was defined according to the American College of Cardiology guidelines [17]. All patients received evidence-based medical therapies including coronary revascularization and statins. Patients were followed up every 3 to 4 months at clinics run by the Hong Kong Hospital Authority. The study was approved by the local Institutional Review Board (IRB approval number: UW18-309) and written informed consent was obtained from all patients. Baseline examinations, outcome classification and sample size calculation details are provided in Additional file 1.

### BP measurements and visit-to-visit BPV

The procedure for measuring BP followed standard guidelines. At each clinic visit, two brachial BPs were measured in the right arm by a nurse using an automated oscillometric device (Dinamap PRO 100) with the patient seated and having rested for 10 min and the mean value recorded. BP measurements taken after the baseline clinic visit and before the occurrence of event of interest or censoring were retrospectively retrieved from patient’s electronic medical record to calculate the mean BP and visit-to-visit BPV. For each patients, mean BP was calculated as the average of the BP readings taken during the follow-up period. Visit-to-visit BPV was defined using coefficient of variation (CV), standard deviation (SD) and average real variability (ARV). All parameters were evaluated for both systolic BP and diastolic BP. Formulas used to calculate the metrics of BPV are presented in Additional file 1.

### Statistical analysis

Continuous data are expressed as mean ± SD and categorical data as number and percentage. The Kolmogorov–Smirnov test was used to evaluate the normality assumption for continuous variables. Any variable with skewed distribution was transformed using natural logarithms prior to analysis. Each BPV measure was categorized by quartile, with the lowest quartile used as the reference. Comparisons between groups were evaluated using Analysis of Variance for continuous variables and Chi-square test for categorical variables. Multivariable linear regression analysis was performed to determine which factors were independently associated with CV of BP. Variables with *P* < 0.10 in the univariable analyses were included in the multivariable linear regression model. Pearson correlation coefficient used to measure the relationship between number of BP measurements and risk factors.

Logistic regression models were used to assess the association of CV of BP with MACE, adjusting for age, gender, clinical risk factors (current smoker, body mass index, T2DM, hypertension), history of myocardial infarction, use of antihypertensive medication (beta-blocker, calcium channel blocker, angiotensin-converting enzyme inhibitor [ACEI] and angiotensin receptor blocker), number of BP measurements, and mean BP level during the same period of BPV measurement. Multivariable models were applied separately for systolic BP and diastolic BP. Details of variable selection for the logistic regression models are described in Additional file 1. Linear trends across quartiles were calculated by including quartile as a continuous variable in the models. Similar analyses were conducted for SD and ARV of systolic and diastolic BP. Tests for the interaction of age, gender and T2DM status with quartile of CV of systolic and diastolic BP were also performed. The incremental predictive value of a model was assessed by area under the receiver operating characteristic curve (AUC), category-free net reclassification index (NRI) and integrated discrimination improvement (IDI). The differences between AUCs were compared using the DeLong test [18].

To account for the number of BP measurements varied between patients, we performed a sensitivity analysis based on BP measurements from the first 6 visits and tested whether the CV calculated from these measurements was associated with MACE. Intraclass correlation coefficient was used to assess the agreement between CV calculated from different number of measurements.

Statistical analyses were performed using STATA statistical software (version 14.0) and R-programming language (version 3.5.3). A two-sided *P* value < 0.05 was considered statistically significant.

## Results

After excluding 73 patients who attended fewer than four clinic visits during the follow-up period, 1140 patients were included in the final analysis and the missing anthropometric data (n = 57) were imputed (Additional file 2: Figure S1). The mean age of study participants was 66 ± 11 years, 75% were male and 402 (35%) had T2DM. The mean number of clinic BP measurements per patient was 21 ± 15 (median, 18), and the median interval between measurements was 132 (interquartile range, 94–173) days. There were 192 (17%) patients experienced a new MACE during the follow-up period (mean 83 months). Among them, 39 (20%) suffered a non-fatal acute myocardial infarction, 31 (16%) developed non-fatal acute coronary syndrome, 39 (20%) had a non-fatal stroke, 12 (6%) had symptomatic peripheral vascular disease requiring treatment, and 71 (37%) died from cardiovascular causes.

Baseline characteristics of all patients with and without MACE are summarized in Table 1. Patients who developed any MACE were significantly older, more likely to be female, and more likely to have hypertension and T2DM, as well as higher mean SD, CV and ARV of systolic and diastolic BP, and a lower baseline diastolic BP than those who did not develop a MACE (all *P* < 0.05). In addition, patients with a MACE were less likely to be prescribed beta-blockers. Nevertheless there were no significant differences in body mass index or lipid levels between those with and without MACE. Compared with non-T2DM patients, patients with T2DM were older, more likely to be female and had a slightly higher body mass index (Additional file 3: Table S1). Also, T2DM patients were more likely to have hypertension and be prescribed ACEIs, and had higher mean, SD, CV and ARV of systolic and diastolic BP.

**Table 1:** Baseline characteristics of the study cohort

Variables	All	Subjects with major adverse cardiovascular events	Subjects without major adverse cardiovascular events	*P* value
n	1140	192	948	
Age, years	66 ± 11	70 ± 9	65 ± 11	< 0.001
Male	859 (75)	133 (69)	726 (77)	0.032
Current smoker	182 (16)	35 (18)	147 (16)	0.35
BMI, kg/m^2^	25.3 ± 3.6	25.3 ± 3.7	25.4 ± 3.6	0.71
T2DM	402 (35)	100 (52)	302 (32)	< 0.001
Hypertension	827 (73)	159 (83)	668 (70)	< 0.001
Prior history of MI	83 (7)	9 (5)	74 (8)	0.13
*Antihypertensive medication*				
ACEI	426 (37)	62 (32)	364 (38)	0.11
ARB	185 (16)	36 (19)	149 (16)	0.30
Beta-blocker	708 (62)	103 (54)	605 (64)	0.008
CCB	369 (32)	62 (32)	307 (32)	0.98
Baseline SBP, mmHg	130.0 ± 17.0	131.3 ± 18.6	129.8 ± 16.6	0.28
Baseline DBP, mmHg	72.6 ± 10.8	69.4 ± 11.7	73.3 ± 10.5	< 0.001
Triglycerides, mmol/L	1.49 ± 1.03	1.53 ± 0.96	1.49 ± 1.04	0.62
Total cholesterol, mmol/L	4.07 ± 0.97	4.09 ± 1.04	4.07 ± 0.95	0.77
HDL cholesterol, mmol/L	1.20 ± 0.33	1.17 ± 0.35	1.21 ± 0.33	0.17
LDL cholesterol, mmol/L	2.16 ± 0.81	2.19 ± 0.88	2.16 ± 0.80	0.58
HbA1c, %	6.59 ± 1.43	6.67 ± 1.41	6.58 ± 1.44	0.39
*Clinic SBP*				
Mean, mmHg	131.3 ± 11.6	133.4 ± 13.9	130.8 ± 11.1	0.004
SD	13.6 ± 4.7	15.7 ± 5.5	13.2 ± 4.4	< 0.001
CV	10.3 ± 3.4	11.8 ± 4.0	10.0 ± 3.2	< 0.001
ARV	14.1 ± 4.9	16.1 ± 5.6	13.7 ± 4.6	< 0.001
*Clinic DBP*				
Mean, mmHg	72.4 ± 8.0	69.9 ± 7.8	73.0 ± 8.0	< 0.001
SD	8.2 ± 2.5	8.9 ± 2.8	8.0 ± 2.4	< 0.001
CV	11.4 ± 3.6	12.9 ± 4.1	11.1 ± 3.4	< 0.001
ARV	8.5 ± 2.8	9.4 ± 3.0	8.3 ± 2.7	< 0.001

Data are shown as mean ± SD or n (%). *ACEI* angiotensin converting enzyme inhibitor, *ARB* angiotensin receptor blocker, *ARV* average real variability, *BMI* body mass index, *CCB* calcium channel blocker, *CV* coefficient of variation, *DBP* diastolic blood pressure, *HbA1c* hemoglobin A1c, *HDL* high-density lipoprotein, *LDL* low-density lipoprotein, *MI* myocardial infarction, *SBP* systolic blood pressure, *SD* standard deviation, *T2DM* type 2 diabetes mellitus

Patients with higher CV of systolic BP were older, more likely to be female, to have T2DM, hypertension and be prescribed calcium channel blockers, and had a lower mean diastolic BP than those with lower CV of systolic BP (Additional file 3: Table S2). Similar associations were found for CV of diastolic BP (Additional file 3: Table S3). Multivariable linear regression analysis showed that CV of systolic BP was significantly and positively associated with age, presence of T2DM and hypertension, and number of BP measurements (Additional file 3: Table S4). Similarly, CV of diastolic BP was significantly and positively associated with age, female gender, presence of hypertension and number of BP measurements, but negatively associated with prescription of beta-blockers and mean diastolic BP. Pearson correlation coefficient showed that the number of BP measurements was correlated with age and the presence of T2DM and hypertension (Additional file 3: Table S5).

In the univariable logistic regression analysis, variables including T2DM, hypertension and beta-blocker use were significantly associated with MACE (all *P* < 0.05) and were entered into the multivariable model (Additional file 3: Table S6). In the multivariable logistic regression model, patients with stable CAD in the highest quartile for CV of systolic BP were at significantly higher risk of MACE compared with those in the lowest quartile, after full adjustment for age, gender, T2DM, hypertension, beta-blocker and ACEI prescription, number of BP measurements and mean systolic BP during the measurement period (Table 2; Model 3). The adjusted odds ratio for the highest versus lowest quartile of CV of systolic BP was 3.30 (95% CI 1.97–5.54; *P* for trend < 0.001). Likewise, the CV of diastolic BP was significantly associated with a higher risk for MACE; the adjusted odds ratio for the highest versus lowest quartile of CV of diastolic BP was 2.39 (95% CI 1.39–4.11; *P* for trend = 0.002). The results were consistent when modeling the variability of systolic and diastolic BP using SD and ARV (Additional file 3: Table S7).

**Table 2:** Odds ratios for MACE associated with quartiles of coefficient of variation of SBP/DBP

	Quartiles of CV				*P* trend
	Q1	Q2	Q3	Q4	
*Systolic blood pressure*					
Events, n (%)	27 (9)	41 (14)	40 (14)	84 (29)	
	OR (95% CI)				
Model 1	1.00 (reference)	1.70 (1.00–2.88)†	1.63 (0.95–2.81)	3.77 (2.27–6.26)*	< 0.001
Model 2	1.00 (reference)	1.59 (0.93–2.72)	1.40 (0.80–2.43)	3.25 (1.94–5.45)*	< 0.001
Model 3	1.00 (reference)	1.60 (0.94–2.75)	1.43 (0.82–2.48)	3.30 (1.97–5.54)*	< 0.001
*Diastolic blood pressure*					
Events, n (%)	26 (9)	41 (14)	55 (19)	70 (25)	
	OR (95% CI)				
Model 1	1.00 (reference)	1.78 (1.03–3.05)†	2.35 (1.38–4.00)*	2.83 (1.68–4.79)*	< 0.001
Model 2	1.00 (reference)	1.72 (0.99–2.99)	2.09 (1.21–3.60)*	2.54 (1.49–4.35)*	0.001
Model 3	1.00 (reference)	1.66 (0.96–2.89)	1.97 (1.13–3.41)†	2.39 (1.39–4.11)*	0.002

Model 1 is adjusted for age, sex and number of BP measurements (ln-transformed)

Model 2 is adjusted for variables in model 1 and type 2 diabetes, hypertension and beta-blockers

Model 3 is adjusted for variables in model 2 and mean SBP or DBP

*CI* confidence interval, *CV* coefficient of variation, *DBP* diastolic blood pressure, *OR* odds ratio, *SBP* systolic blood pressure

^†^*P* < 0.05; *P < 0.01

The risk factor model (composed by covariates in Model 2) yielded an AUC of 0.70 (95% CI 0.65–0.74) for prediction of MACE (Table 3). The addition of systolic or diastolic CV to the risk factor model with mean BP significantly improved the model performance, with AUC further increased to 0.73 (95% CI 0.69–0.77; difference in AUCs, 0.03; *P* = 0.002) for systolic BP and 0.72 (95% CI 0.68–0.76; difference in AUCs, 0.02; *P* = 0.007) for diastolic BP. There was no significant increase in AUC when only mean systolic or diastolic BP was added to the risk factor model (Table 3; Model S1/D1). As shown in Table 3, significant improvement in risk reclassification and discrimination was also observed for models with the addition of systolic or diastolic CV (Model S2: NRI, 26.3%; 95% CI 10.9–41.8; IDI, 2.3%; 95% CI 1.0–3.4; Model D2: NRI, 25.2%; 95% CI 9.8–40.7; IDI, 1.6%; 95% CI 0.5–2.8).

**Table 3:** Performance of models predicting MACE in stable CAD patients

Model	Feature	AUC (95% CI)	*P* value†	*P* value*	NRI (95% CI)	*P* value	IDI (95% CI)	*P* value
Risk factor model	Age + Male + T2DM + Hypertension + Beta-blockers + Number of BP measurements	0.69 (0.65–0.74)	Reference	–	–		–	
Model S1	Risk factor model + Mean SBP	0.70 (0.65–0.74)	0.80	Reference	9.4 (− 6.1 to 24.9)	0.23	0.1 (− 0.1 to 0.4)	0.24
Model S2	Model S1 + CV of SBP	0.73 (0.69–0.77)	0.002	0.002	26.3 (10.9–41.8)	< 0.001	2.3 (1.0–3.4)	< 0.001
Model D1	Risk factor model + Mean DBP	0.70 (0.66–0.74)	0.18	Reference	13.8 (− 1.7 to 29.2)	0.080	0.4 (− 0.1 to 0.9)	0.13
Model D2	Model D1 + CV of DBP	0.72 (0.68–0.76)	0.007	0.024	25.2 (9.8–40.7)	0.001	1.6 (0.5–2.8)	0.004

Values for number of BP measurements were ln-transformed before analysis. *AUC* area under the curve, *CI* confidence interval, *CV* coefficient of variation, *DBP* diastolic blood pressure, *IDI* integrated discrimination improvement, *NRI* net reclassification index, *SBP* systolic blood pressure, *T2DM* type 2 diabetes mellitus

^†^*P* value from DeLong test for comparison of AUCs; *Comparison of AUCs between model S2 versus S1 and model D2 versus D1

Interaction analyses showed that the association of CV of systolic and diastolic BP with occurrence of MACE differed between patients with and without T2DM (*P*_interaction_, 0.027 for systolic CV; 0.035 for diastolic CV). No significant interaction of age or gender with CV of systolic and diastolic BP (all *P*_interaction_ > 0.05) was observed in the multivariable models.

Patients with T2DM had a higher rate of MACE than those without (25% versus 12%; *P* < 0.001). After full multivariable adjustment (Model 3), higher CV of systolic BP remained significantly associated with MACE in both patients with and without T2DM, whereas a significant association of CV of diastolic BP with risk of MACE was observed only in patients without T2DM (Table 4).

**Table 4:** Adjusted odds ratios for MACE in patients with and without T2DM

	Quartiles of CV				*P* trend
	Q1	Q2	Q3	Q4	
	OR (95% CI)				
*Systolic blood pressure*					
T2DM	1.00 (reference)	1.27 (0.61–2.64)	1.43 (0.69–2.95)	2.41 (1.19–4.87)†	0.013
No T2DM	1.00 (reference)	1.08 (0.49–2.40)	2.10 (1.00–4.41)	3.41 (1.67–6.96)*	< 0.001
*Diastolic blood pressure*					
T2DM	1.00 (reference)	1.48 (0.72–3.04)	1.20 (0.56–2.59)	1.82 (0.89–3.73)	0.15
No T2DM	1.00 (reference)	0.90 (0.41–2.01)	1.85 (0.89–3.85)	2.20 (1.06–4.58)†	0.006

All models adjusted for age, sex, hypertension, beta-blockers, number of BP measurements (ln-transformed) and mean SBP or DBP

*CI* confidence interval, *CV* coefficient of variation, *DBP* diastolic blood pressure, *OR* odds ratio, *SBP* systolic blood pressure, *T2DM* type 2 diabetes mellitus

^†^*P* < 0.05; *P < 0.01

The sensitivity analysis showed that the association of BPV with MACE among patients with stable CAD was similar and remained significant when based on CV of BP calculated from 6 measurements (Additional file 3: Table S8).

In the intraclass correlation coefficient analysis, the BPV calculated from 6 measurements showed good agreement (intraclass correlation coefficient, 0.83 for systolic CV; 0.80 for diastolic CV) with the BPV calculated from 12 measurements. The CV of systolic and diastolic BP increased when more BP measurements were added into the calculation (Additional file 3: Table S9).

## Discussion

Our results provide further evidence of a strong association between visit-to-visit systolic and diastolic BPV and MACE in patients with established CAD. These associations persisted after further multivariable adjustment including mean BP and was consistent across sensitivity analyses. Moreover, there was significant interaction of T2DM status with systolic and diastolic BPV. Higher systolic BPV was associated with an increased risk of MACE in both patients with and without T2DM, whereas the association of diastolic BPV with MACE was confined to patients without T2DM. The risk associated with systolic and diastolic BPV was stronger among those without T2DM than with T2DM. The addition of systolic or diastolic BPV to a model with conventional risk factors and mean BP provided incremental benefit for predicting MACE in patients with stable CAD. These results provide novel insight into the impact of T2DM on the prognostic value of BPV beyond mean BP and conventional risk factors in CVD risk prediction for patients with CAD.

Evidence of the prognostic value of BPV in CVD remains weak. In a recent report from the ADVANCE-ON study, Ohkuma et al. showed that the addition of visit-to-visit systolic BPV to a model with traditional risk factors and mean systolic BP significantly improved CVD risk prediction in patients with T2DM [19]. In another study of 2157 patients with a history of CVD, addition of systolic BPV yielded only a modest improvement in CVD risk prediction [20]. To the best of our knowledge, ours is the first study to demonstrate that visit-to-visit variability of both systolic and diastolic BP provide incremental predictive values for future cardiovascular events beyond mean BP and conventional risk factors in patients with stable CAD with or without T2DM.

High systolic BPV has been shown to be associated with cardiovascular comorbidities and an independent predictor of cardiovascular events and mortality, particularly in patients with established CAD, stroke or T2DM [21–23]. In this study, we have demonstrated that the highest quartile of CV of systolic BP was significantly associated with a 2.41-fold higher risk of MACE than the lowest quartile in CAD patients with T2DM. Importantly, this association was more pronounced among patients without T2DM: the highest quartile of CV of systolic BP was associated with a 3.41-fold higher risk than the lowest quartile. These findings suggest that systolic BPV is important in patients at different levels of baseline risk and could be used to identify high-risk patients for preventive interventions.

Some studies have reported that visit-to-visit diastolic BPV was also positively associated with risk for CVD events [23, 24], but others have reported contradictory findings [25, 26]. It has been suggested that BPV is influenced by number of visits and the time between visits as well as other factors such as method used to estimate BPV and duration of follow-up, and that this may explain the inconsistent results [13, 27]. In the present study, we have shown that diastolic BPV was independently associated with MACE in CAD patients without T2DM, but not in those with T2DM. Indeed, recent meta-analysis involving 377,305 patients with T2DM also found no association between long-term diastolic BPV and adverse cardiovascular outcomes or mortality [28], and is consistent with our results. These findings might explain the conflicting results for the predictive value of diastolic BPV for CVD events since patients with and without T2DM were included [23, 24]. Nonetheless the exact mechanisms underlying the relationship between diastolic BPV and cardiovascular events in patients with stable CAD and T2DM remain unclear. Studies have demonstrated that increased BPV is associated with development or progression of diabetic nephropathy [29]. It has also been suggested that increased BPV is an early indication of autonomic dysfunction in patients with T2DM [30]. Cardiovascular autonomic neuropathy is a severe complication of diabetes and associated with increased cardiovascular mortality and morbidity [31]. The risk markers for cardiovascular autonomic neuropathy in patients with T2DM include age, obesity, hypertension, duration of diabetes, glycemic control and development of other microvascular complications [32]. In our study cohort, patients with T2DM were older and had a higher prevalence of hypertension and higher body mass index, suggesting a high potential risk for development of autonomic dysfunction. Thus, the link between autonomic dysfunction and increased BPV may have influenced the association between diastolic BPV and cardiovascular outcomes in patients with and without T2DM.

The mechanisms underlying the link between visit-to-visit BPV and adverse cardiovascular events are not fully understood. In this study, CAD patients with higher quartile of systolic and diastolic BPV were associated with those higher risk features in terms of elderly age and higher prevalence of hypertension and diabetes than those in the lowest quartiles, despite of only a few mmHg differences in their baseline systolic and diastolic blood pressure. These findings suggest that higher BPV is a better predictor to identify patients with clustering of high risk factors for vascular aging, and thus increase subsequent MACEs. Indeed, increased visit-to-visit BPV has been associated with increased arterial stiffness, a marker of vascular aging, and strong predictor of cardiovascular events [33, 34]. Increasing value of BPV has also been shown to be associated with a higher prevalence and severity of target organ damage such as diastolic dysfunction, and inversely associated with aortic distensibility and arterial elasticity [35]. Recent studies have also found significant associations between BPV and markers of endothelial dysfunction and inflammation [36]. Impaired baroreflex sensitivity and autonomic dysfunction have also been reported to be associated with increased BPV [30, 37]. Further studies are needed to better understand the mechanistic relationship between long-term BPV and cardiovascular outcomes.

In real-world clinical practice, the number of office BP measurements varies in individuals, and those with only a few BP readings may result in greater visit-to-visit BPV. To date, the number of visits needed for defining visit-to-visit BPV have not standardized. In the present study, we found that the increase in risk associated with BPV was only slightly attenuated but remain statistically significant when using 6 measurements to calculate visit-to-visit BPV. Similar results were found when based on BPV calculated from 12 measurements (data not shown). In addition, we found high concordance between BPV calculated from 12 measurements and BPV calculated from 6 or 8 measurements, suggesting that a shorter duration of measurements could potentially be used for monitoring BPV. A recent study has also shown similar levels of concordance when compare the BPV derived from 20 measurements with the BPV from 6 or more measurements, and proposed to use a minimum of 6 measurements for a reliable estimation of visit-to-visit BPV [38].

Our study has several limitations. First, we did not obtain information on the dosage of antihypertensive drug or patient compliance with treatment, both of which have been shown to contribute to BPV [39]. Second, we cannot conclude that there is a causal relationship between long-term BPV and development of cardiovascular events. Third, potential confounders such as severity of CAD, glycemic and lipid control, antidiabetic drug use and duration of diabetes were not available in the present study. Fourth, it is possible that changes to antihypertensive medication regimen during the follow-up period may have affected the association between long-term BPV and outcomes, and should be further investigated. Finally, larger studies are required to confirm our findings in CAD patients with and without T2DM.

## Conclusions

Visit-to-visit variabilities of systolic BP and diastolic BP are independent predictors of new MACE in patients with stable CAD. Moreover, systolic and diastolic BPV provide additional predictive information beyond mean BP and conventional risk factors for future cardiovascular events. The association of BPV in CAD patients without T2DM with subsequent risk of MACE is stronger than in those with T2DM. Our findings support the potential utility of visit-to-visit BPV in risk stratification and management of high-risk patients.

## Supplementary Information

**Additional file 1.** Supplemental Methods

**Additional file 2: Figure S1.** Flow chart of study recruitment. BP, blood pressure; BPV, blood pressure variability; CAD, coronary artery disease.

**Additional file 3: Table S1.** Comparison of baseline characteristics in patients with and without T2DM. **Table S2.** Baseline characteristics according to quartiles of coefficient of variation of SBP. **Table S3.** Baseline characteristics according to quartiles of coefficient of variation of DBP. **Table S4.** Multivariable linear regression models for coefficient of variation of SBP/DBP. **Table S5.** Pearson correlation coefficient analysis based on number of BP measurements. **Table S6.** Logistic regression analysis for predicting MACE in stable CAD patients. **Table S7.** Odds ratios for MACE associated with quartiles of SD and ARV of SBP/DBP. **Table S8.** Odds ratios for MACE associated with CV of SBP/DBP, based on six BP measurements. **Table S9.** Intraclass correlation coefficient for CV of SBP/DBP.

## Data Availability

The datasets used and/or analyzed during the current study are available from the corresponding author on reasonable request.

## Abbreviations

ACEI: Angiotensin-converting enzyme inhibitor
ARV: Average real variability
AUC: Area under the curve
BP: Blood pressure
BPV: Blood pressure variability
CAD: Coronary artery disease
CI: Confidence interval
CV: Coefficient of variation
CVD: Cardiovascular disease
OR: Odds ratio
IDI: Integrated discrimination improvement
MACE: Major adverse cardiovascular events
NRI: Net reclassification index
SD: Standard deviation
T2DM: Type 2 diabetes mellitus
