# Supplementary material for: Predictive value of visit-to-visit blood pressure variability for cardiovascular events in patients with coronary artery disease with and without diabetes mellitus
Source: Cardiovasc Diabetol. 2021 Apr 24;20:88. doi: 10.1186/s12933-021-01280-z (PMC8070286; doi:10.1186/s12933-021-01280-z)
Supplement: Supplementary file 1 — Additional file 1. Supplemental Methods [file 12933_2021_1280_MOESM1_ESM.docx]

**Supplemental Methods**

**Formulas used to calculate the metrics of visit-to-visit BPV**

$$Standard deviation (SD)=\sqrt{\frac{{\sum(x_{i}-\bar{x})}^{2}}{n-1}}$$

$$Coefficient of variation=\frac{SD}{\bar{x}_{i}}\times100\%$$

$$Average real variability=\frac{1}{n-1}\sum_{i=1}^{n-1} \left| {BP}_{i+1}-{BP}_{i} \right|$$

where *n* is the number of valid BP measurements of a given participant.

**Baseline examinations**

Demographic data, medical history, family history, medication use, smoking status, blood pressure, and anthropometric measurements including body weight and height were recorded at enrollment. Missing anthropometric data were imputed using the multivariate imputation method in the R package MICE (multivariate imputations by chained equations) [1]. Hypertension referred to the presence of a history of hypertension or regular prescription of antihypertensive medication. T2DM was defined according to World Health Organization 1998 diagnostic criteria or regular prescription of antidiabetic medication [2]. Current smokers were defined as individuals who reported using any tobacco product in the last 30 days. Blood samples were drawn from patients at recruitment after a 12-hour overnight fast to measure glucose and lipid profile.

**Outcomes**

The primary endpoint was the occurrence of a new MACE during the study period. The diagnosis of MACE was according to the International Classification of Diseases, Ninth Revision (ICD-9) and included acute myocardial infarction (ICD-9 410), acute coronary syndrome (ICD-9 411.1), stroke (ICD-9 430, 431, 433, 434, 436), peripheral vascular disease (ICD-9 443.9), and cardiovascular death (death certificate ICD-9 410-447). Information on the date of events and discharge diagnosis were verified from medical records of the Hong Kong Hospital Authority database. Data on deaths including the primary cause and date of death were obtained from the Hong Kong Death Registry.

**Sample size calculation**

Based on the findings from a previous study [3], and assuming the prediction model with BPV and clinical risk factor would detect a hazard ratio of 1.72 for cardiovascular outcomes, 138 events were required to provide 80% power with a two-sided significance level of 0.05. Effective sample size was achieved based on the rule of ten outcome events per variable for the Cox regression-based prediction model [4].

**Statistical analysis**

*Variable selection for logistic regression models*

Model 1 adjusted for age, gender and number of BP measurements (to account for the possibility that the number of measurements might influence BPV); model 2 further adjusted for variables associated with MACE in the univariable models with *P*<0.10; model 3: the fully adjusted model, further adjusted for mean systolic or diastolic BP as appropriate.

**References**

1. Buuren S, Groothuis-Oudshoorn K. MICE: Multivariate imputation by chained equations in R. Journal of statistical software. 2011;45(3)

2. Alberti KG, Zimmet PZ: Definition, diagnosis and classification of diabetes mellitus and its complications. Part 1: diagnosis and classification of diabetes mellitus provisional report of a WHO consultation. *Diabet Med* 1998, 15(7):539-553.

3. Vidal-Petiot E, Stebbins A, Chiswell K, Ardissino D, Aylward PE, Cannon CP, Ramos Corrales MA, Held C, Lopez-Sendon JL, Stewart RAH *et al*: Visit-to-visit variability of blood pressure and cardiovascular outcomes in patients with stable coronary heart disease. Insights from the STABILITY trial. *Eur Heart J* 2017, 38(37):2813-2822.

4. Vittinghoff E, McCulloch CE: Relaxing the rule of ten events per variable in logistic and Cox regression. *Am J Epidemiol* 2007, 165(6):710-718.
