# Supplementary figures and images for: Predictive value of visit-to-visit blood pressure variability for cardiovascular events in patients with coronary artery disease with and without diabetes mellitus
Source: Cardiovasc Diabetol. 2021 Apr 24;20:88. doi: 10.1186/s12933-021-01280-z (PMC8070286; doi:10.1186/s12933-021-01280-z)

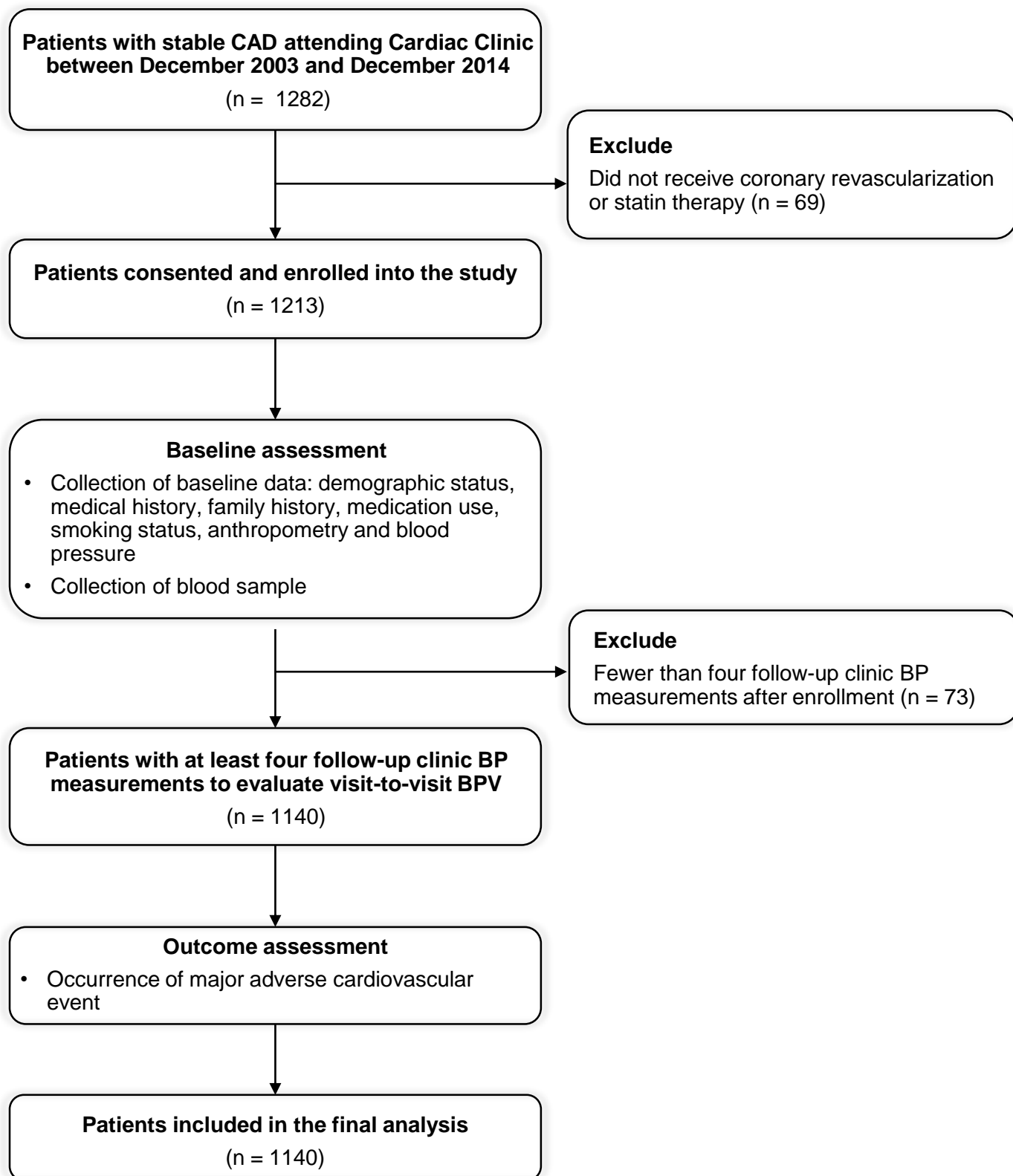

Supplement: Supplementary file 2 — Additional file 2: Figure S1. Flow chart of study recruitment. BP, blood pressure; BPV, blood pressure variability; CAD, coronary artery disease. [file 12933_2021_1280_MOESM2_ESM.pdf]
