# Supplementary material for: Predictive value of visit-to-visit blood pressure variability for cardiovascular events in patients with coronary artery disease with and without diabetes mellitus
Source: Cardiovasc Diabetol. 2021 Apr 24;20:88. doi: 10.1186/s12933-021-01280-z (PMC8070286; doi:10.1186/s12933-021-01280-z)
Supplement: Supplementary file 3 — Additional file 3: Table S1. Comparison of baseline characteristics in patients with and without T2DM. Table S2. Baseline characteristics according to quartiles of coefficient of variation of SBP. Table S3. Baseline characteristics according to quartiles of coefficient of variation of DBP. Table S4. Multivariable linear regression models for coefficient of variation of SBP/DBP. Table S5. Pearson correlation coefficient analysis based on number of BP measurements. Table S6. Logistic regression analysis for predicting MACE in stable CAD patients. Table S7. Odds ratios for MACE associated with quartiles of SD and ARV of SBP/DBP. Table S8. Odds ratios for MACE associated with CV of SBP/DBP, based on six BP measurements. Table S9. Intraclass correlation coefficient for CV of SBP/DBP. [file 12933_2021_1280_MOESM3_ESM.docx]

| **Table S1. Comparison of baseline characteristics in patients with and without T2DM** | | | |
| --- | --- | --- | --- |
| Variables | T2DM | No T2DM | *P* value |
| n | 402 | 738 |  |
| Age, years | 67±10 | 66±11 | 0.040 |
| Male | 288 (72) | 571 (77) | 0.032 |
| Current smoker | 66 (16) | 116 (16) | 0.76 |
| BMI, kg/m^2^ | 25.7±3.6 | 25.2±3.5 | 0.030 |
| Hypertension | 352 (88) | 475 (64) | <0.001 |
| Prior history of MI | 22 (5) | 61 (8) | 0.083 |
| Antihypertensive medication | |  |  |
| ACEI | 168 (42) | 258 (35) | 0.023 |
| ARB | 70 (17) | 115 (16) | 0.42 |
| Beta-blocker | 254 (63) | 454 (62) | 0.58 |
| CCB | 136 (34) | 233 (32) | 0.44 |
|  |  |  |  |
| Clinic SBP |  |  |  |
| Mean, mmHg | 132.9±11.7 | 130.4±11.5 | <0.001 |
| SD | 14.76±4.8 | 13.0±4.5 | <0.001 |
| CV | 11.0±3.4 | 10.0±3.3 | <0.001 |
| ARV | 14.8±4.7 | 13.7±4.9 | <0.001 |
| Clinic DBP |  |  |  |
| Mean, mmHg | 71.0±7.5 | 73.2±8.2 | <0.001 |
| SD | 8.5±2.5 | 8.0±2.5 | <0.001 |
| CV | 12.1±3.5 | 11.0±3.6 | <0.001 |
| ARV | 8.7±2.8 | 8.4±2.8 | 0.047 |
| Data are shown as mean ± SD or n (%). Abbreviations: ACEI, angiotensin converting enzyme inhibitor; ARB, angiotensin receptor blocker; ARV, average real variability; BMI, body mass index; CCB, calcium channel blocker; CV, coefficient of variation; DBP, diastolic blood pressure; MI, myocardial infarction; SBP, systolic blood pressure; SD, standard deviation; T2DM, type 2 diabetes mellitus. | | | |

| **Table S2. Baseline characteristics according to quartiles of coefficient of variation of SBP** | | | | | |
| --- | --- | --- | --- | --- | --- |
|  | Quartiles of SBP CV | | | | *P* value |
| Variables | Q1 (<8.15%) | Q2 (8.15 to <9.90%) | Q3 (9.90 to <12.14%) | Q4 (≥12.14%) |  |
| n | 285 | 285 | 285 | 285 |  |
| Age, years | 63±10 | 64±11 | 67±10 | 70±10 | <0.001 |
| Male | 234 (82) | 221 (78) | 204 (72) | 200 (70) | 0.003 |
| Current smoker | 33 (12) | 47 (16) | 55 (19) | 47 (16) | 0.087 |
| BMI, kg/m^2^ | 25.4±3.5 | 25.6±3.4 | 25.4±3.5 | 25.0±3.9 | 0.19 |
| T2DM | 60 (21) | 93 (33) | 125 (44) | 124 (44) | <0.001 |
| Hypertension | 177 (62) | 199 (70) | 211 (74) | 240 (84) | <0.001 |
| Prior history of MI | 29 (10) | 20 (7) | 19 (7) | 15 (5) | 0.14 |
| Antihypertensive medication | |  |  |  |  |
| ACEI | 96 (34) | 119 (42) | 96 (34) | 115 (40) | 0.081 |
| ARB | 46 (16) | 50 (18) | 50 (18) | 39 (14) | 0.56 |
| Beta-blocker | 186 (65) | 187 (66) | 169 (59) | 166 (58) | 0.14 |
| CCB | 75 (26) | 90 (32) | 95 (33) | 109 (38) | 0.024 |
|  |  |  |  |  |  |
| Clinic blood pressures |  |  |  |  |  |
| Mean SBP, mmHg | 131.0±11.2 | 131.2±12.7 | 130.4±10.9 | 132.3±11.6 | 0.26 |
| Mean DBP, mmHg | 75.4±7.8 | 73.1±7.5 | 71.0±8.2 | 70.3±7.6 | <0.001 |
| Data are shown as mean ± SD or n (%). Abbreviations: ACEI, angiotensin converting enzyme inhibitor; ARB, angiotensin receptor blocker; BMI, body mass index; CCB, calcium channel blocker; CV, coefficient of variation; DBP, diastolic blood pressure; HbA1c, hemoglobin A1c; HDL, high-density lipoprotein; LDL, low-density lipoprotein; MI, myocardial infarction; SBP, systolic blood pressure; T2DM, type 2 diabetes mellitus. | | | | | |

| **Table S3. Baseline characteristics according to quartiles of coefficient of variation of DBP** | | | | | |
| --- | --- | --- | --- | --- | --- |
|  | Quartiles of DBP CV | | | | *P* value |
| Variables | Q1 (<8.90%) | Q2 (8.90 to <10.99%) | Q3 (10.99 to <13.55%) | Q4 (≥13.55%) |  |
| n | 285 | 285 | 285 | 285 |  |
| Age, years | 62±10 | 65±10 | 68±10 | 70±10 | <0.001 |
| Male | 236 (83) | 233 (82) | 200 (70) | 190 (67) | <0.001 |
| Current smoker | 42 (15) | 50 (18) | 47 (16) | 43 (15) | 0.78 |
| BMI, kg/m^2^ | 25.4±3.2 | 25.4±3.5 | 25.4±3.6 | 25.2±4.0 | 0.92 |
| T2DM | 65 (23) | 98 (34) | 122 (43) | 117 (41) | <0.001 |
| Hypertension | 179 (63) | 196 (69) | 222 (78) | 230 (81) | <0.001 |
| Prior history of MI | 32 (11) | 21 (7) | 14 (5) | 16 (6) | 0.018 |
| Antihypertensive medication | |  |  |  |  |
| ACEI | 105 (37) | 110 (39) | 105 (37) | 106 (37) | 0.97 |
| ARB | 36 (13) | 47 (17) | 59 (21) | 43 (15) | 0.066 |
| Beta-blocker | 195 (68) | 182 (64) | 175 (61) | 156 (55) | 0.008 |
| CCB | 71 (25) | 84 (29) | 107 (38) | 107 (38) | 0.002 |
|  |  |  |  |  |  |
| Clinic blood pressures |  |  |  |  |  |
| Mean SBP, mmHg | 130.5±11.0 | 130.1±11.5 | 131.7±11.7 | 132.7±12.2 | 0.031 |
| Mean DBP, mmHg | 76.2±7.8 | 73.0±7.2 | 70.7±7.5 | 69.9±8.1 | <0.001 |
| Data are shown as mean ± SD or n (%). Abbreviations: ACEI, angiotensin converting enzyme inhibitor; ARB, angiotensin receptor blocker; BMI, body mass index; CCB, calcium channel blocker; CV, coefficient of variation; DBP, diastolic blood pressure; HbA1c, hemoglobin A1c; HDL, high-density lipoprotein; LDL, low-density lipoprotein; MI, myocardial infarction; SBP, systolic blood pressure; T2DM, type 2 diabetes mellitus. | | | | | |

| **Table S4. Multivariable linear regression models for coefficient of variation of SBP/DBP** | | | | |
| --- | --- | --- | --- | --- |
|  | SBP CV | | DBP CV | |
|  | Standardized β-coefficient | *P* value | Standardized β-coefficient | *P* value |
| Age | 0.236 | <0.001 | 0.191 | <0.001 |
| Male | -0.037 | 0.20 | -0.066 | 0.021 |
| T2DM | 0.074 | 0.012 | 0.053 | 0.063 |
| Hypertension | 0.080 | 0.010 | 0.086 | 0.006 |
| Prior history of MI | 0.004 | 0.88 | -0.014 | 0.62 |
| Beta-blocker | -0.051 | 0.075 | -0.085 | 0.002 |
| CCB | 0.017 | 0.56 | 0.047 | 0.096 |
| Mean DBP | - |  | -0.174 | <0.001 |
| Number of BP measurements | 0.186 | <0.001 | 0.141 | <0.001 |
| Values for number of BP measurements were ln-transformed before analysis. Abbreviations: ACEI, angiotensin converting enzyme inhibitor; ARB, angiotensin receptor blocker; CCB, calcium channel blocker; CV, coefficient of variation; DBP, diastolic blood pressure; MI, myocardial infarction; SBP, systolic blood pressure; T2DM, type 2 diabetes mellitus. | | | | |

| **Table S5. Pearson correlation coefficient analysis based on number of BP measurements** | | |
| --- | --- | --- |
|  | r | *P* value |
| Age | -0.11 | <0.001 |
| Male | 0.02 | 0.52 |
| Current smoker | 0.01 | 0.72 |
| BMI | 0.05 | 0.11 |
| T2DM | 0.20 | <0.001 |
| Hypertension | 0.11 | <0.001 |
| Mean SBP | -0.03 | 0.36 |
| Mean DBP | -0.05 | 0.064 |
| Values for number of BP measurements were ln-transformed before analysis. Abbreviations: BMI, body mass index; DBP, diastolic blood pressure; SBP, systolic blood pressure; T2DM, type 2 diabetes mellitus. | | |

| **Table S6. Logistic regression analysis for predicting MACE in stable CAD patients** | | |
| --- | --- | --- |
| Variables | Unadjusted OR (95% CI) | *P* value |
| Age | 1.05 (1.04-1.07) | <0.001 |
| Male | 0.69 (0.49-0.97) | 0.033 |
| Current smoker | 1.21 (0.81-1.82) | 0.35 |
| BMI | 0.99 (0.95-1.04) | 0.71 |
| T2DM | 2.33 (1.70-3.18) | <0.001 |
| Hypertension | 2.02 (1.35-3.01) | 0.001 |
| Prior history of MI | 0.58 (0.29-1.18) | 0.13 |
| Antihypertensive medication | |  |
| ACEI | 0.77 (0.55-1.06) | 0.11 |
| ARB | 1.24 (0.83-1.85) | 0.30 |
| Beta-blocker | 0.66 (0.48-0.9) | 0.008 |
| CCB | 1.00 (0.71-1.39) | 0.98 |
| Abbreviations: ACEI, angiotensin converting enzyme inhibitor; ARB, angiotensin receptor blocker; BMI, body mass index; CCB, calcium channel blocker; CI, confidence interval; OR, odds ratio; MI, myocardial infarction; T2DM, type 2 diabetes mellitus. | | |

| **Table S7. Odds ratios for MACE associated with quartiles of SD and ARV of SBP/DBP** | | | | | | |
| --- | --- | --- | --- | --- | --- | --- |
|  |  | Quartiles of BP variability measure | | | | *P* trend |
| Variability parameter |  | Q1 | Q2 | Q3 | Q4 |  |
| SBP SD | Events, n (%) | 27 (9) | 31 (11) | 52 (18) | 82 (29) |  |
|  |  | OR (95% CI) | | | |  |
|  | Model 3 | 1.00 (reference) | 1.12 (0.64-1.97) | 1.86 (1.09-3.18)† | 3.08 (1.81-5.25)* | <0.001 |
|  |  |  |  |  |  |  |
| SBP ARV | Events, n (%) | 30 (11) | 29 (10) | 49 (17) | 84 (29) |  |
|  |  | OR (95% CI) | | | |  |
|  | Model 3 | 1.00 (reference) | 0.96 (0.55-1.67) | 1.57 (0.93-2.64) | 2.57 (1.57-4.19)* | <0.001 |
|  |  |  |  |  |  |  |
| DBP SD | Events, n (%) | 37 (13) | 37 (13) | 50 (18) | 68 (24) |  |
|  |  | OR (95% CI) | | | |  |
|  | Model 3 | 1.00 (reference) | 0.94 (0.56-1.58) | 1.38 (0.84-2.28) | 1.93 (1.19-3.15)* | 0.002 |
|  |  |  |  |  |  |  |
| DBP ARV | Events, n (%) | 30 (11) | 39 (14) | 52 (18) | 71 (25) |  |
|  |  | OR (95% CI) | | | |  |
|  | Model 3 | 1.00 (reference) | 1.31 (0.77-2.23) | 1.84 (1.1-3.07)† | 2.55 (1.55-4.2)* | <0.001 |
| Model 3 is adjusted for age, sex, number of BP measurements (ln-transformed), type 2 diabetes, hypertension, beta-blockers and mean SBP or DBP | | | | | | |
| Abbreviations: ARV, average real variability; CI, confidence interval; DBP, diastolic blood pressure; OR, odds ratio; SBP, systolic blood pressure; SD, standard deviation. | | | | | | |
| †*P*<0.05; **P*<0.01 | | | | | | |

| **Table S8. Odds ratios for MACE associated with CV of SBP/DBP, based on six BP measurements** | | | |
| --- | --- | --- | --- |
|  | Events (%)  (n = 1107) | Adjusted OR (95% CI) | *P* trend |
| SBP CV |  |  |  |
| Q1 | 22 (13) | 1.00 (reference) | <0.001 |
| Q2 | 32 (19) | 1.43 (0.80-2.55) |  |
| Q3 | 44 (74) | 1.96 (1.13-3.41)† |  |
| Q4 | 74 (43) | 3.13 (1.85-5.29)* |  |
|  |  |  |  |
| DBP CV |  |  |  |
| Q1 | 24 (14) | 1.00 (reference) | 0.035 |
| Q2 | 44 (26) | 1.78 (1.04-3.06)† |  |
| Q3 | 43 (25) | 1.68 (0.98-2.89) |  |
| Q4 | 61 (35) | 1.93 (1.13-3.28)* |  |
| All models adjusted for age, sex, type 2 diabetes, hypertension, beta-blockers and mean SBP or DBP | | | |
| Abbreviations: CI, confidence interval; CV, coefficient of variation; DBP, diastolic blood pressure; OR, odds ratio; SBP, systolic blood pressure. | | | |
| †*P*<0.05; **P*<0.01 | | | |

| **Table S9. Intraclass correlation coefficient for CV of SBP/DBP** | | | | | | |
| --- | --- | --- | --- | --- | --- | --- |
|  | SBP CV | | DBP CV | | Duration for completing the number of BP measurements | Number of patients |
| BP measurement | Mean±SD | ICC (95% CI) | Mean±SD | ICC (95% CI) | Median (IQR) | n |
| 4 | 9.1±4.5 | 0.71 (0.64-0.75)* | 9.7±4.9 | 0.65 (0.57-0.71)* | 14 (9-18) | 1140 |
| 6 | 9.4±3.9 | 0.83 (0.80-0.86)* | 10.0±4.3 | 0.80 (0.75-0.83)* | 22 (15-30) | 1107 |
| 8 | 9.6±3.7 | 0.92 (0.90-0.93)* | 10.3±3.8 | 0.92 (0.90-0.93)* | 30 (21-40) | 1039 |
| 12 | 10.1±3.2 | Reference | 10.7±3.4 | Reference | 42 (29-53) | 755 |
| Abbreviations: CI, confidence interval; CV, coefficient of variation; DBP, diastolic blood pressure; ICC, intraclass correlation coefficient; SBP, systolic blood pressure. | | | | | | |
| **P*<0.01 | | | | | | |
